# MassTRIX Reloaded: Combined Analysis and Visualization of Transcriptome and Metabolome Data

**DOI:** 10.1371/journal.pone.0039860

**Published:** 2012-07-06

**Authors:** Brigitte Wägele, Michael Witting, Philippe Schmitt-Kopplin, Karsten Suhre

**Affiliations:** 1 Institute of Bioinformatics and Systems Biology, Helmholtz Zentrum München, German Research Center for Environmental Health, Neuherberg, Germany; 2 Department of Genome oriented Bioinformatics, Life and Food Science Center Weihenstephan, Technische Universität München, Freising-Weihenstephan, Germany; 3 Research Unit Analytical BioGeoChemistry, Helmholtz Zentrum München, German Research Center for Environmental Health, Neuherberg, Germany; 4 Chair of Analytical Food Chemistry, Technische Universität München, Freising-Weihenstephan, Germany; 5 Department of Physiology and Biophysics, Weill Cornell Medical College in Qatar, Education City–Qatar Foundation, Doha, Qatar; University of Toronto, Canada

## Abstract

Systems Biology is a field in biological science that focuses on the combination of several or all “omics”-approaches in order to find out how genes, transcripts, proteins and metabolites act together in the network of life. Metabolomics as analog to genomics, transcriptomics and proteomics is more and more integrated into biological studies and often transcriptomic and metabolomic experiments are combined in one setup. At a first glance both data types seem to be completely different, but both produce information on biological entities, either transcripts or metabolites. Both types can be overlaid on metabolic pathways to obtain biological information on the studied system. For the joint analysis of both data types the MassTRIX webserver was updated. MassTRIX is freely available at www.masstrix.org.

## Introduction

In Systems Biology, Metabolomics is an interesting field as end-point of the paradigm that biological information flows from the genome over the transcriptome and proteome to the metabolome; it is the sum of all biological processes, including post translational modification and regulations. To generate hypotheses about regulatory processes between different levels, “omics”-approaches are combined in one experimental setup, whereas the combination of transcriptomics and metabolomics is often preferred. Gene expression data can nowadays be obtained by the use of gene expression chips or more recently with Next-Generation-Sequencing of transcripts. Since usually gene expression correlates well with translation, it reflects the protein levels to certain degree. Additionally, modern mass spectrometry (MS) technology is able to detect thousands of metabolites with high accuracy and precision. In particular, Ion Cyclotron Resonance Fourier Transform MS (ICR-FT/MS) and the newest generation of Time of Flight MS (ToF/MS) provide mass errors <0.1 ppm or <2 ppm, respectively. This accuracy, together with isotopic information allows the calculation of chemical formulas following several chemical rules [1]. The derived chemical formulas can be searched against databases, but by themselves formulas alone deliver no or less biological information. Visualization of measured data in a biological context is the most practical first step in analyzing such data sets. Several solutions for visualization of “omics”-data already exist. PATHOS, for example, allows the mapping of MS data to metabolic pathways [2]. Another example is the Paintomics webserver, which allows the joint visualization of already identified and pre-analyzed data from metabolomics and transcriptomics experiments [3]. Despite the fact that these tools are useful for visualization, none of the mentioned applications are able to analyze data from both transcriptomics and metabolomics from the scratch. The updated version of MassTRIX presented here allows a combined analysis and visualization of metabolomic and transcriptomic data, including raw data from Affymetrix Genechips, in one setup.

## Methods

**Figure 1:**
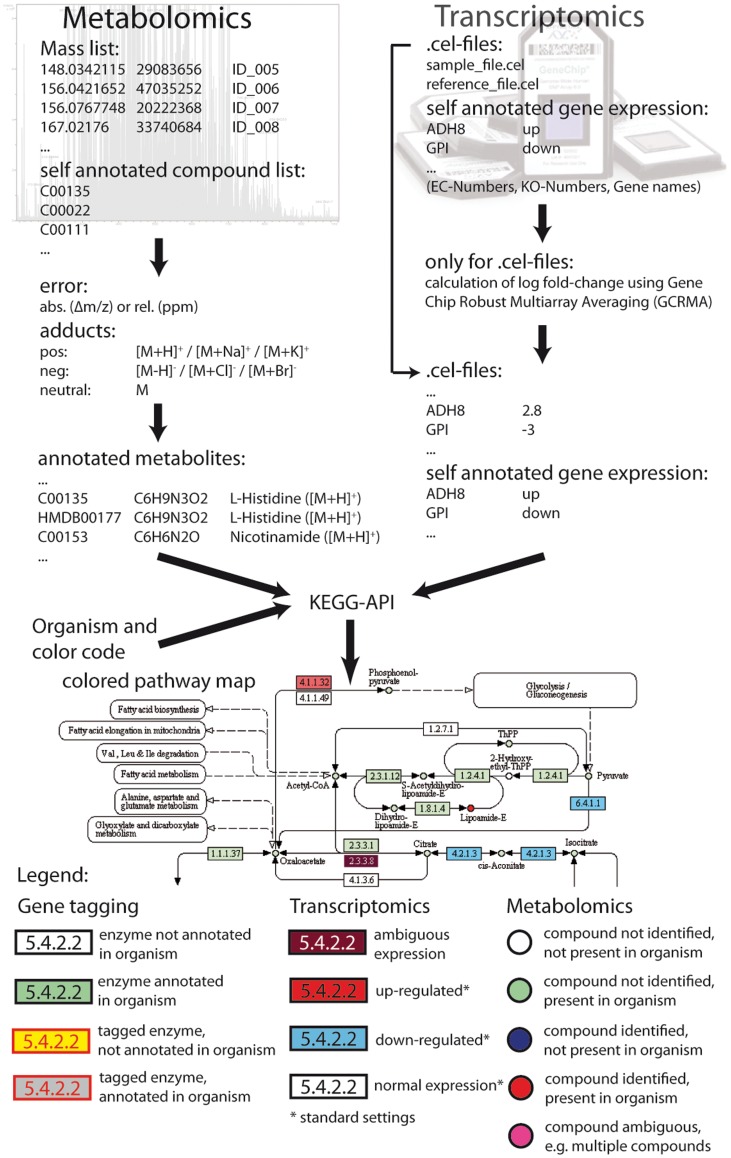
Representation of the systematic workflow of MassTRIX for annotation of MS and transcriptomic data. Both data types are combined and submitted to KEGG via the KEGG API to obtain colored pathway maps. The coloring for transcriptomic data can be defined by the user.

The MassTRIX webserver is written in Perl using CGI for dynamic content representation and runs on an Apache2 web server (version 2.2.11).

**Figure 2:**
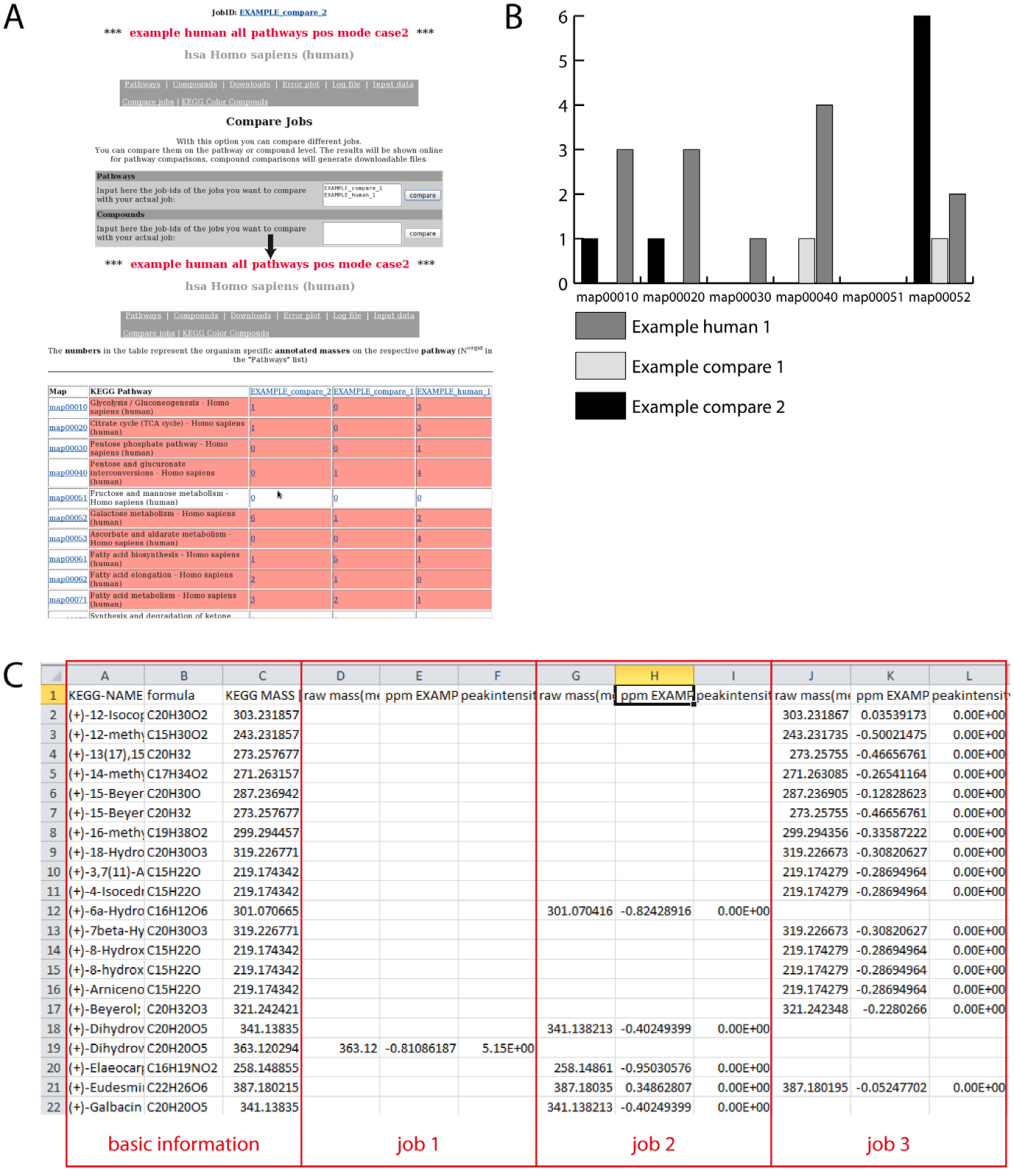
Possible comparisons between jobs in MassTRIX. (A) Screen shot from the “Compare jobs” functionality and the obtained result page comparing jobs on pathway level. (B) Result from comparing 3 different jobs on pathway level represented in a barplot. X-Axis represents the different pathway maps and Y-Axis the number of annotated compounds on this pathway. (C) Result from comparing 3 different jobs on compound level. The file can directlybe downloaded and opened in MS Excel.

A calibrated mass list, consisting of tab separated masses, intensity values and an additional unique identifier, like an ID or a retention time, serves as main input for MassTRIX. These masses are compared within a certain error range against the theoretical adducts of compounds from different metabolic databases. The default database is a combination of KEGG [4], [5], HMDB [6] and LipidMaps [7] with isotopic peaks. As alternatives the same combination without isotopic peaks, KEGG expanded lipids (where all residues R in formulas, are exchanged by hydrocarbon chains of different lengths), LipidMaps alone for lipidomics and MetaCyc [8] as other databases or a separate m/z list are possible. For the analysis of gene expression data from Affymetrix arrays, R (version 2.10) with the gcrma package are used. Affymetrix identifiers are mapped to the respective KEGG ontology (KO) numbers for visualization on metabolic pathway maps. Correctly annotated KEGG metabolites and KO’s are colored on the respective pathways of a chosen organism by calling the KEGG API. Additionally, enzymes of interest, submitted either as EC-numbers or KEGG identifiers can be highlighted. The obtained maps are fully clickable and cross-linked between the different result pages. Different jobs can be compared on pathway or compound level and all results are downloadable.

## Results

The full basic functionality of MassTRIX explained in Suhre and Schmitt-Kopplin [9] is preserved, but several new features are added in the updated version. For positive ionization mode potassium ad-ducts ([M+K]^+^) and for negative ionization chloride ([M+Cl]^−^) and bromine ([M+Br]^−^) adducts, including the most relevant isotopes, are added. Isotopic peaks are filtered according to the natural abundance, meaning that masses corresponding to isotopes are only kept if the main adduct peak is found. For example an [M+^37^Cl]^−^ adduct is only kept if also the corresponding [M+^35^Cl]^−^ is also found. An exception from this is bromine, having a natural distribution of both isotopes of roughly 50%. Here peaks are kept only if both isotopes are found. By default only [M+H]^+^ or [M-H]^−^ in the respective ionization mode are used for the search. The web interface allows the specification of either a relative ppm or absolute Da error. The absolute error scale enables the use with MS instruments of lower resolution, like Time-of-Flight instruments. Instead of a mass list, a file containing KEGG compound numbers can be uploaded, bypassing the whole annotation procedure for already identified metabolites. As several metabolites and molecules from different organisms are not listed in certain databases, we included the possibility to upload a separate database in flat file format containing a list of molecules (as pre-calculated adducts) which will be also searched. This also allows to create a list of adducts not covered by MassTRIX, e.g. [M+H-H_2_O]^+^ or [M-2H]^2−^. A comprehensive overview on commonly observed ESI adducts can be found in Huang et al. [10]. If KEGG ID’s are provided with this list, they can be also colored in the pathways. The general workflow of MassTRIX is shown in Figure 1.

As new feature transcriptomic data can be uploaded. MassTRIX accepts the.cel format of Affymetrix, a self-annotated file or gene expression data pasted into the data field for data input. For gene expression with cel-files a reference file as control and a sample file are uploaded and are analyzed via GC robust multi array averaging using an R script. A self-annotated file or data to paste contains either a EC-numbers, KEGG identifiers or KO’s and tab separated from them a fold change or UP or DOWN as key word for the gene expression analysis. All processed data is visualized on the corresponding KEGG pathways of a chosen organism via the KEGG API, whereas by default only the “Glycolysis and Gluconeogenesis” pathway is used, saving time, since most users use MassTRIX for annotation of MS data. If other pathways should be included, it needs to be pointed out explicitly by uploading a list of pathways to be colored. It should be noted that even if only the “Glycolysis and Gluconeogenesis” pathway is chosen, information on the metabolite-pathway relationship of all pathways is included in the downloadable files.

All results are downloadable as tab separated text files. The masses.annotated file includes all annotated raw masses with the respective information, including theoretical adduct mass, ppm or absolute error, number of possible isomers/isobars, KEGG ID’s, molecular formula and pathway information. A second compressed format is available containing all possible annotations for a raw mass together in one line with the additional metabolite-pathway relationship. Moreover files with the used input and option settings or fold changes together with png images of the colored pathways can be downloaded.

For further analysis, results from the metabolomics part can be compared between jobs on either the compound or the pathway level. This feature enables the comparison of different experimental conditions or multiple time points in an experimental setup. The comparison of pathways can be used for deducing which pathways are affected by a treatment or sample condition compared to a control. It is carried out with the pathway number, also allowing comparison between different organisms. Pathways not present in one organism but in another will also be considered, whereby the number of annotated compounds is empty in the case of non-presence. Compound comparison uses information stored in the masses.annotated file and creates a matrix of all annotated compounds from all used jobs against the different jobs. For each job three columns, containing the submitted intensity, the calculated ppm error and the raw mass, are included. Metabolites not found in a certain job are left empty. An example can be found in Figure 2.

Different combinations of the upload possibilities can be tested with the example jobs on the MassTRIX webserver.

## Discussion

The updated version of MassTRIX allows the combined visualization of gene expression and metabolome data in one setup. Moreover the ability to compare different jobs on pathway or metabolite levels enables the analysis of time-series or experiments under different conditions.

Current limitations are based on the insufficiency of the KEGG API in regards to multi-coloring of pathways and multiplexing of different samples on a shared representation. Despite this impediment, KEGG normally allows a good first overview on biology before using species specific databases, were available. The inclusion of MetaCyc gives the possibility to annotate metabolites stored in this database, being more specific for bacterial genomes and pathways [8].

This updated version fulfills the requirements of scientists working with both transcriptomic and metabolomic data. It allows for a quick overview of their data in the biological context of metabolic pathways and therefore enables the generation of first hypotheses for further in-depth analysis.

Further improvements are ongoing with the integration of additional metabolic databases, e.g. KNApSAcK [11] and the effort to catch up with growing field of meta-genomics and meta-metabolomics, not only analyzing single isolated species, but involving the concept of superorganisms with whole communities like ocean or gut microbiomes.
